# Effect of Static Magnetic Field on *Monascus ruber* M7 Based on Transcriptome Analysis

**DOI:** 10.3390/jof7040256

**Published:** 2021-03-30

**Authors:** Shuyan Yang, Hongyi Zhou, Weihua Dai, Juan Xiong, Fusheng Chen

**Affiliations:** 1 Hubei International Scientific and Technological Cooperation Base of Traditional Fermented Foods, Huazhong Agricultural University, Wuhan 430070, China; shuyanyang@webmail.hzau.edu.cn (S.Y.); zhouhongyi1@newhope.cn (H.Z.); dwh@webmail.hzau.edu.cn (W.D.); 2 College of Food Science and Technology, Huazhong Agricultural University, Wuhan 430070, China; 3 College of Science, Huazhong Agricultural University, Wuhan 430070, China; xiong@mail.hzau.edu.cn

**Keywords:** *Monascus ruber*, static magnetic field, *Monascus* pigments, citrinin, transcriptomic analysis

## Abstract

The effects of a static magnetic field (SMF) on *Monascus ruber* M7 (*M. ruber* M7) cultured on potato dextrose agar (PDA) plates under SMF treatment at different intensities (5, 10, and 30 mT) were investigated in this paper. The results revealed that, compared with the control (CK, no SMF treatment), the SMF at all tested intensities did not significantly influence the morphological characteristics of *M. ruber* M7, while the intracellular and extracellular *Monascus* pigments (MPs) and extracellular citrinin (CIT) of *M. ruber* M7 were increased at 10 and 30 mT SMF but there was no impact on the MPs and CIT at 5 mT SMF. The transcriptome data of *M. ruber* M7 cultured at 30 mT SMF on PDA for 3 and 7 d showed that the SMF could increase the transcriptional levels of some relative genes with the primary metabolism, including the carbohydrate metabolism, amino acid metabolism, and lipid metabolism, especially in the early growing period (3 d). SMF could also affect the transcriptional levels of the related genes to the biosynthetic pathways of MPs, CIT, and ergosterol, and improve the transcription of the relative genes in the mitogen-activated protein kinase (MAPK) signaling pathway of *M. ruber* M7. These findings provide insights into a comprehensive understanding of the effects of SMF on filamentous fungi.

## 1. Introduction

*Monascus* spp., is a type of filamentous fungi, and its fermented product, Hongqu, also known as red yeast, Anka or red mold rice, has been used and produced in China and other Asian countries for nearly 2000 years [1,2]. *Monascus* spp. has received worldwide attention because it produces abundant beneficial secondary metabolites (SMs) [3], such as the well-known monacolin K (MK, also called lovastatin), an inhibitor of cholesterol biosynthesis [4]; γ-aminobutyric acid with hypotensor effects [5]; ergosterol (ERG), a precursor of vitamin D_2_ [6]; and *Monascus* pigments (MPs), used as natural food coloring agents [7]. However, the discovery of citrinin (CIT) [8], a nephrotoxic mycotoxin produced by certain strains of *Monascus* spp., gave rise to controversy over the safety of Hongqu.

Magnetic fields (MFs) are ubiquitous environmental factors that markedly affect the growth, development, and behavior of many species of organisms [9,10,11,12]. Researchers often develop artificial MFs (hereinafter referred to as MFs) since the geomagnetic field cannot be adjusted and its intensity is weak [13]. MFs can be divided into two categories: the static magnetic field (SMF) generated by a permanent magnet or direct current passing through a metal coil, whose north and south poles are typically unchanged in the same experiment [9]; and the alternative magnetic field (AMF) generated by an alternating current through the metal coil, whose north and south poles change with the frequency of the alternating current [14]. The effects of various types of MFs on the growth and metabolism of microorganisms have mainly involved bacteria [15,16] and yeasts [17,18]. For example, exposure of *Komagataeibacter xylinus* ATCC 53524 to 50 Hz AMF resulted in an increase in bacterial cellulose yield and a drop in fructose consumption [19]. An SMF at 206.3 mT enhanced the dye decolorization efficiency and halotolerance of *Pichia occidentalis* A2 [20].

Recently, the MF effects, including SMF and low-frequency AMF (LF-AMF, <300 Hz) on filamentous fungi were studied. Ahmad et al. found that the SN-pole (between the southern and northern poles) of SMF inhibited the concentration of total aflatoxin produced by *Aspergillus flavus* [21]. Mateescu et al. discovered that 0.62 T SMF inhibits the growth of *A. niger* [22], while another study showed that LF-AMF increased the yield of citric acid and cellulase activity produced by *A. niger* [23]. LF-AMF also increased the yields of MPs [24,25] and MK [26], and inhibited CIT production [27] in *M. purpureus*. The regulatory mechanisms underlying the effect of LF-AMF on MPs and CIT have been explored at the protein level [26]; however, the effects of SMF on *Monascus* spp. at the molecular level are not available.

The genome of *Monascus ruber* M7 (*M. ruber* M7) has been sequenced, and the biosynthetic pathways of MPs and CIT in *M. ruber* M7 have been well studied in our laboratory [7,28], showing that it can produce MPs and CIT but not MK [29]. In this study, the SMF effects on *M. ruber* M7 were analyzed through transcriptomics combining with morphological characteristics and yields of MPs and CIT. The results may provide some clues to elucidate the mechanism of MF on organisms.

## 2. Materials and Methods

### 2.1. Static Magnetic Field Device and Its Treatment on M. ruber M7

**Figure 1:**
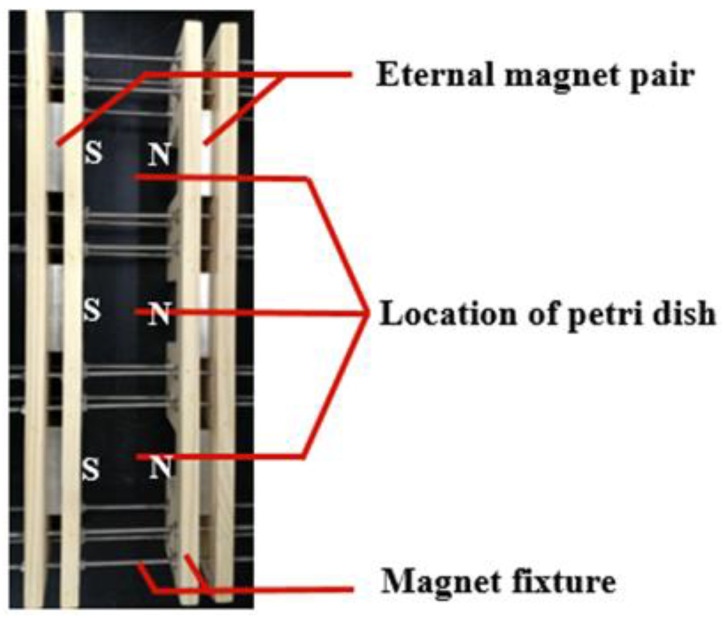
The device of static magnetic field produced by a pair of permanent magnets [30].

The static magnetic field (SMF) device was set up in our laboratory (Figure 1), in which a pair of permanent magnets was clamped with fixtures consisting of wooden plates and screws to form a magnet pair, and the magnetic flux densities could be controlled by adjusting the distance of two magnets [30]. The magnetic densities were measured using a Gauss meter (SJ700, Senjie Technology Co., Ltd., Guilin, China). The PDA plates inoculated with *M. ruber* M7 were put under different intensities (5, 10, and 30 mT) of SMF.

### 2.2. Strains and Culture Conditions

*M. ruber* M7 (CCAM 070120) stored in our laboratory [7] was cultivated on a PDA slant at 28 °C for 12 days, and its spore suspension (adjusted to 10^5^ spores/mL) was prepared with sterile water.

### 2.3. Morphological Analysis of M. ruber M7

We inoculated 100 μL of the spore suspension of M7 prepared in Section 2.1 in the center of PDA plates to observe its colonial morphologies; meanwhile, 200 μL on the spore suspension were evenly spread on the PDA plates, and sterile coverslips were inserted into the media at 45° obliquely to analyze the microscopic morphologies [31]. The PDA plates were kept under 5, 10, and 30 mT SMF at 28 °C, with no SMF treatment for CK. The morphological characters of M7 were observed on the 4th and 9th days.

### 2.4. Determination of MPs, CIT and ERG Produced by M. ruber M7

Two hundred μL of freshly harvested spore suspension of *M. ruber* M7 prepared in Section 2.1 were spread onto plates (Φ = 9 cm) containing 20 mL PDA, covered with cellophane, and incubated at 28 °C for 9 days under 5, 10, and 30 mT SMF. The mycelia and media were collected every 2 days from 3 to 9 days and freeze-dried to detect the MPs, CIT, and EGR contents.

For MPs and CIT detection, 0.1 g of freeze-dried mycelia (for intracellular MPs and CIT) or media powder (for extracellular MPs and CIT) was suspended in 3 mL 80% (*v/v*) methanol solution, subjected to 30 min of ultrasonication treatment (KQ-250B, Kunshan, China), followed by centrifugation at 10,000× *g* for 5 min to collect the supernatants. The extraction was repeated once, and the supernatants were merged. The optical density (OD) values of the combined supernatant were measured at 505 nm using a UV−vis spectrophotometer (UV-1700, Shimadzu, Tokyo, Japan). The total OD_505nm_ values were regarded as the MPs content, and one OD value was taken as one MPs unit (U) [32]. The supernatant was used to analyze the CIT content by high-performance liquid chromatography (HPLC, LC-20AT, Shimadzu, Tokyo, Japan) following the established method in our laboratory [33].

For EGR detection, 0.05 g of freeze-dried mycelia powder was suspended in absolute ethanol following the extraction method from Chang et al. [34] to detect the ERG content using HPLC. The ERG was separated by an Inertsil ODS-3 C18 column (250 mm × 4.6 mm, 5 μm), using isocratic elution with 100% methanol as the mobile phase and a flow rate of 1.0 mL/min.

### 2.5. Transcriptome Analysis of M. ruber M7 Treated with 30 mT SMF

*M. ruber* M7 on PDA plates covered with cellophane membranes was put under 30 mT SMF at 28 °C, with no SMF treatment for CK. Fresh mycelia after cultured 3 and 7 d were harvested to extract the total RNA, respectively, which was sequenced using the BGIseq-500RS platform (BGI, Wuhan, China, http://en.genomics.cn/ accessed on 15 February 2021). The expression levels of nine randomly selected genes in *M. ruber* M7 under 30 mT SMF at the 7th day were validated by quantitative real-time PCR (qRT-PCR) to confirm the reliability of the transcriptome results, with *β-actin* which was not affected by SMF serving as the reference gene.

The raw data obtained by sequencing were counted by the software SOAPnuke, then filtered with Trimmomatic to remove low-quality reads and obtain clean reads for analysis. The clean reads were compared with the *M. ruber* M7 genome [2] and the gene sequences of *M. ruber* M7 by hierarchical indexing for spliced alignment of transcripts (HISAT) [35] and Bowtie2 [36], respectively.

The gene expression levels were estimated with RNA-Seq by Expectation-Maximization (RSEM) [37]. The normalized value of fragments per kilobase of transcript per million mapped reads (FPKM) was used as a parameter to compare the expression levels between CK and the experimental groups. Differential expression analysis of two groups was performed using the DEseq2 package [38]. Genes with a fold change (FC) ≥ 1.5 (|log_2_FC| ≥ 0.584963) and *Q* value (adjusted *p*-value) ≤ 0.05 were selected as differentially expressed genes (DEGs) [39].

Kyoto Encyclopedia of Genes and Genomes (KEGG) pathway (https://www.kegg.jp/ accessed on 15 February 2021) function and enrichment analyses were implemented to investigate the functions of the DEGs. The DEGs involved in the primary metabolism, secondary metabolism, and signal transduction pathways were also analyzed to explore the SMF effect mechanisms on *M. ruber* M7.

### 2.6. Statistical Analysis

The data were statistically analyzed using analysis of variance (ANOVA) for a completely randomized block design with SPSS 22.0 software (SPSS Inc., Chicago, IL, USA), and the differences in means were determined using the least significant differences (LSD). Three biological replicates were used for each treatment, and the results are expressed as the mean ±  standard deviation of the number of experiments. *p*-values less than 0.05 were considered as statistically significant.

## 3. Results

### 3.1. Effects of SMF on the Morphological Characteristics of M. ruber M7

The morphological characteristics of *M. ruber* M7 treated under different SMF densities (5, 10, and 30 mT) on PDA media were observed to investigate the SMF influences on M7. The results (Supplementary Figure S1) revealed that, compared with CK, all SMF treatment groups had no significant difference in the colonic and microbiological morphologies, which could normally produce conidia and cleistothecia.

### 3.2. Effects of SMF on MPs, and CIT Produced by M. ruber M7

**Figure 2:**
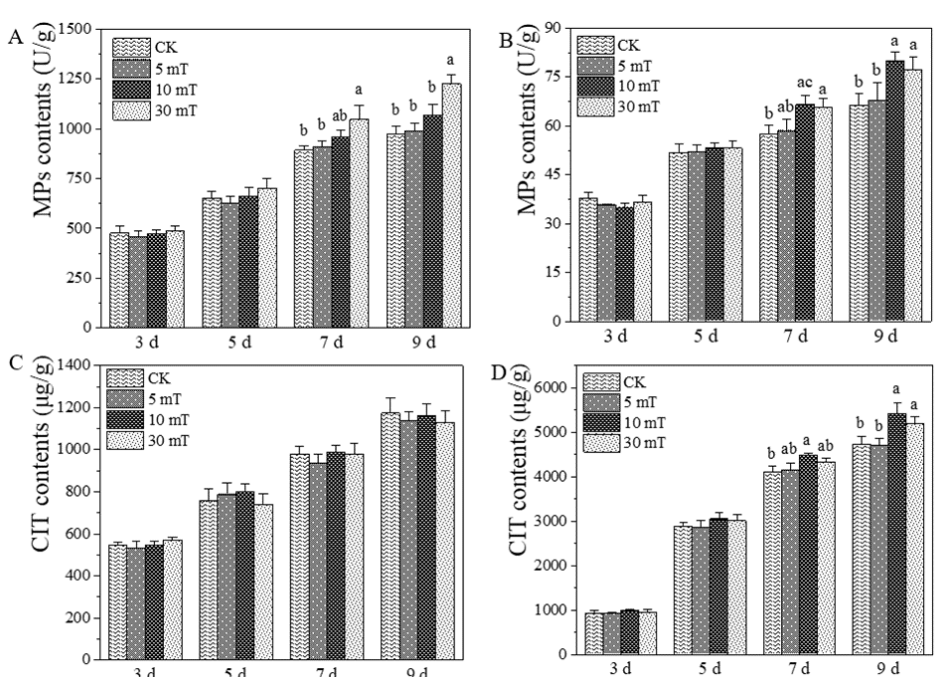
*Monascus* pigments (MPs) and citrinin (CIT) produced by *M. ruber* M7 under the static magnetic field. (**A**). The intracellular MPs; (**B**). The extracellular MPs; (**C**). The intracellular CIT; (**D**). The extracellular CIT. The error bars indicate the standard deviations of three independent cultures. Lowercase letters signify a *p*-value < 0.05.

The SMF effects on MPs and CIT produced by *M. ruber* M7 were determined. The results showed that SMF increased the accumulation of intracellular (in-)/extracellular (ex-) MPs and ex-CIT mainly at the late growth stages (7 and 9 d) of *M. ruber* M7 (Figure 2). At 9 d, compared with CK, the in-MPs production level of M7 under 30 mT SMF was increased by 26.3%, and the ex-MPs production levels under 10 mT and 30 mT SMF were increased by 20.5% and 16.5%, respectively, while the ex-CIT production levels of M7 under 10 mT and 30 mT SMF were increased by 14.7% and 9.8%, respectively. However, all tested SMFs in the current research had no significant impact on the in-CIT contents, as with the 5 mT SMF on the contents of in-/ex-MPs and ex-CIT. Overall, the 30 mT SMF had the stronger effects on the MPs and CIT production; therefore, this SMF treatment group was chosen for subsequent transcriptomic analysis to explore the mechanisms of SMF on *M. ruber* M7.

### 3.3. The Transcriptomic Analysis of M. ruber M7 under 30 mT SMF

**Figure 3:**
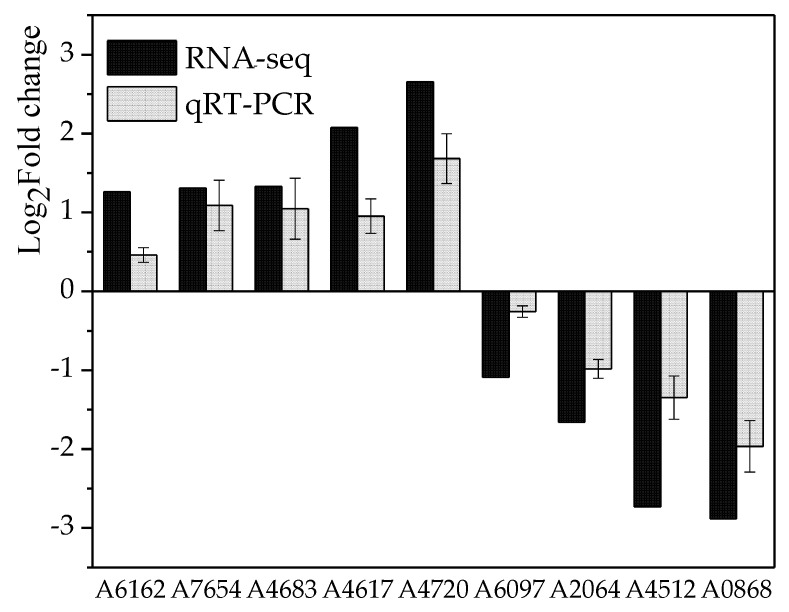
Comparison of the gene expression levels from transcriptomic data and qRT-PCR.

The high-throughput sequencing technology was used to investigate the effect of 30 mT SMF on the transcript levels of *M. ruber* M7 at the 3rd and 7th days. After quality control and data filtering, the GC percentage of the sequencing data, average quality scores more than a 30 reading (Q30) percentage, genome mapping ratios, and gene mapping ratios are shown in Table 1. To evaluate the quality of the transcriptomic data, nine genes in the *M. ruber* M7 genome [2] under 30 mT SMF at the 7th day were randomly selected to analyze their expression levels by qRT-PCR with *β-actin* as the reference gene, with the primer sequences shown in Supplementary Table S1. The results revealed that the qRT-PCR data of the nine selected genes had the same trends as ones in the transcriptomic data of *M. ruber* M7 (Figure 3). All the above results indicated that the accuracy and quality of the transcriptomic data of *M. ruber* M7 were sufficient for further analysis.

#### 3.3.1. Analysis of Differentially Expressed Genes

**Figure 4:**
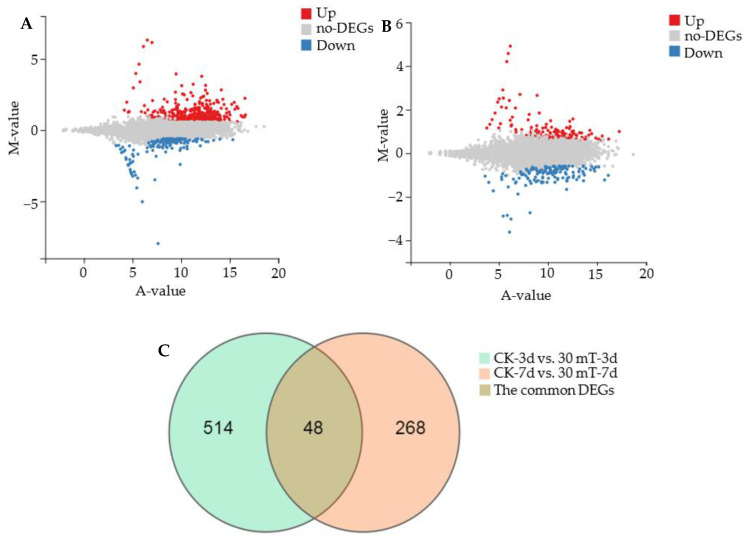
Differentially expressed genes (DEGs) in *M. ruber* M7 under 30 mT SMF. (**A**): *MA*-plot (M-versus-A plot) analysis of the DEGs in the CK-3d vs. 30 mT-3d group. The A-value on the *X*-axis represents the gene expression level calculated by log_2_, and the M-value on the *Y*-axis represents the gene difference multiple calculated by log_2_; (**B**): *MA*-plot analysis of DEGs in CK-7d vs. 30 mT-7d group; (**C**): Venn diagram analysis of the DEGs.

The genes with a fold change ≥1.5 and *Q* value ≤ 0.05 were selected as differentially expressed genes (DEGs). As shown in the *MA*-plots (M-versus-A plot) [40], the numbers of up-regulated and down-regulated DEGs in the CK-3d vs. 30 mT-3d group were 413 and 149, respectively (Figure 4A), while the DEGs in CK-7d vs. the 30 mT-7d group were 173 up-regulated and 143 down-regulated (Figure 4B). In the Venn diagram (Figure 4C), the numbers of common DEGs in CK-3d vs. 30 mT-3d and CK-7d vs. 30 mT-7d groups were only 48. The results of the DEGs (Figure 4) indicate that the gene transcriptional levels in *M. ruber* M7 at 30 mT SMF were diverse in different culture periods and that a stronger up-regulation of the genes in *M. ruber* M7 existed in the early growing period (3 d).

#### 3.3.2. The KEGG Pathways and Their Enrichment Analyses for DEGs

**Figure 5:**
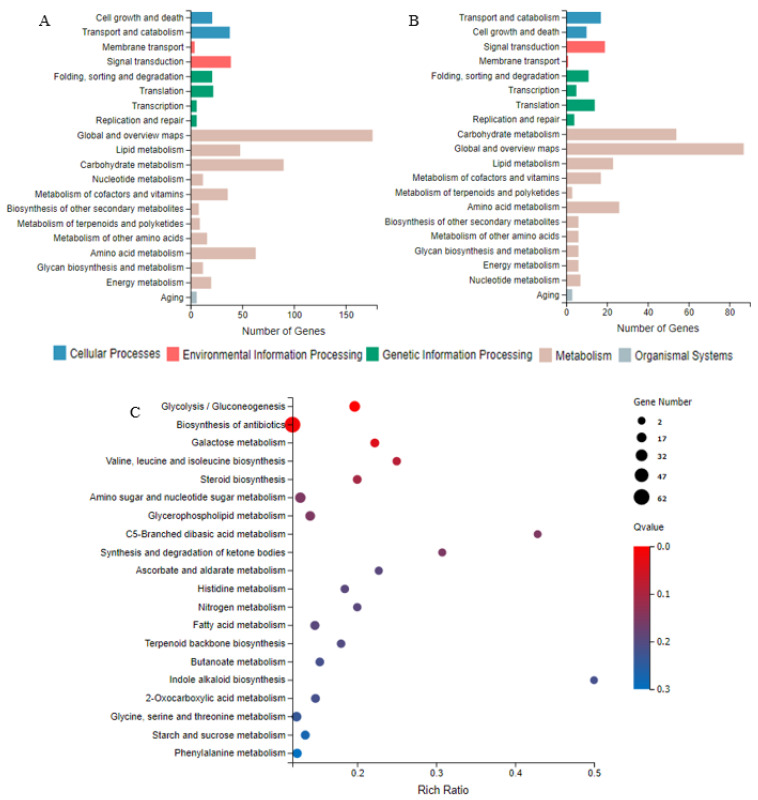
KEGG pathways and their enrichment analyses of DEGs in *M. ruber* M7 cultivated at 3 and 7 d under 30 mT SMF. (**A**): KEGG pathway of DEGs of CK-3d vs. 30 mT-3d; (**B**): KEGG pathways of DEGs of CK-7d vs. 30 mT-7d; (**C**): KEGG pathway enrichment DEGs of CK-3d vs. 30 mT-3d. The enrichment ratio indicates the number of DEGs relative to the percentage of all annotated genes involved in the pathway.

The DEGs’ functions between CK-3d vs. 30 mT-3d and CK-7d vs. 30 mT-7d were investigated through the KEGG pathways. As shown in Figure 5A,B, among five KEGG categories, the most abundant DEGs in different culture periods (3 and 7d) were metabolism, followed by genetic information processing, cellular processes, environmental information processing and organismal systems. In addition, in the KEGG subcategories, the DEGs were mainly involved in global and overview maps, carbohydrate metabolism, amino acid metabolism, lipid metabolism, signal transduction and transport and catabolism, etc. The DEGs of CK-3d vs. 30 mT-3d were significantly enriched in metabolism-related pathways, such as glycolysis/gluconeogenesis and the galactose metabolism, as well as in biosynthesis of antibiotics pathways as demonstrated with KEGG pathway enrichment analysis (Figure 5C). However, the DEGs of CK-7d vs. 30 mT-7d had no significantly enriched KEGG pathways, with the *Q* value > 0.05 (Supplementary Table S2).

#### 3.3.3. Analysis of the DEGs Involved in the Primary Metabolism

According to the KEGG pathways (Figure 5), 30 mT SMF had a wide range of effects on the transcription levels of the related genes with the primary metabolisms, such as the carbohydrate, amino acid, and lipid metabolisms in *M. ruber* M7 at different culture periods (3 and 7 d).

DEGs related to the carbohydrate metabolisms are shown in Supplementary Table S3. In the early growing period (3 d), 24 DEGs (20 DEGs up-regulated/4 DEGs down-regulated, hereinafter referred to as 20/4), 10 DEGs (8/2), and 11 DEGs (9/2) were found in the glycolysis/gluconeogenesis pathway, in the galactose metabolism pathway, and in the starch and sucrose metabolism pathway, respectively. In the late growing period (7 d), 9 DEGs (4/5), 3 DEGs (0/3), and 10 DEGs (4/6) were found in the glycolysis/gluconeogenesis pathway, in the galactose metabolism pathway, and in the starch and sucrose metabolism pathway, respectively. In general, 30 mT SMF mainly influenced the carbohydrate metabolism by positively regulating the transcription levels of the DEGs related to the glycolysis/gluconeogenesis pathway in the early growing period (3 d), while in the late growing period (7 d), 30 mT SMF had an impact on the carbohydrate metabolism by regulating the transcription levels of DEGs related to the starch and sucrose metabolism pathway.

DEGs related to the amino acid metabolism are shown in Supplementary Table S4. We found that 30 mT SMF could affect the biosynthesis and metabolism of various amino acids. In the early growing period (3 d), 13 DEGs (9/4), 11 DEGs (7/4), 16 DEGs (9/7), and 7 DEGs (5/2) were discovered in the phenylalanine metabolism pathway; in the tyrosine metabolism pathway; in the glycine, serine, and threonine metabolism pathway; and in the valine, leucine, and isoleucine biosynthesis pathway, respectively. In the late growing period (7 d), 9 DEGs (3/6), 10 DEGs (2/8), and 2 DEGs (1/1) were found in the phenylalanine metabolism pathway; in the tyrosine metabolism pathway; and in the glycine, serine, and threonine metabolism pathway, respectively. In conclusion, SMF had a stronger regulatory effect on the phenylalanine and tyrosine metabolism in different culture periods (3 and 7 d).

DEGs related to the lipid metabolism are shown in Supplementary Table S5. In the early growing period (3 d), 16 DEGs (13/3), 12 DEGs (8/4), and 10 DEGs (6/4) were found in the glycerophospholipid metabolism pathway; in the fatty acid metabolism; and in the fatty acid degradation pathway, respectively. In the late growing period (7 d), 8 DEGs (6/2), 2 DEGs (1/1), and 4 DEGs (1/3) were found in the glycerophospholipid metabolism pathway; in the fatty acid metabolism pathway; and in the fatty acid degradation pathway, respectively. Overall, SMF had a strong positive regulatory impact on the lipid metabolism-related pathways in the early growing period (3 d).

#### 3.3.4. Analysis of the DEGs Involved in the Secondary Metabolism

The transcript levels of genes related to the biosynthesis of the main secondary metabolites MPs, CIT, and ERG in *M. ruber* M7 under 30 mT SMF were investigated, and the results are shown in Supplementary Table S6.

In the MPs and CIT biosynthesis gene clusters, the transcript levels of related genes were different in the different growth periods of *M. ruber* M7 under SMF treatment. In the early growing period (3 d), only *MpigL* was up-regulated while *MpigD*, *MpigG*, and *MpigN* were down-regulated in the MPs gene cluster. *MRR5* and *MRR6* were up-regulated, while *MRL3*, *MRL5*, *MRL6*, and *MRL7* were down-regulated in the CIT gene cluster. In the late growing period (7 d), *MpigA*, *MpigG*, and *MpigM* in the MP gene cluster were significantly up-regulated as well as four DEGs, including *MRL6*, *MRL5*, *MRL2*, and *MRR1*, in the CIT gene cluster. The transcription levels of seven genes related to the ERG biosynthesis were significantly improved in the early growing period (3 d), while the transcriptional levels of three genes were significantly up-regulated in the late growing period (7 d). Overall, SMF had a strong positive regulatory effect on the transcriptional levels of the relative genes with ERG biosynthesis in the early growing period.

In general, the results of transcriptomic analysis showed that SMF not only affected the production of MPs and CIT but may also affect the production of ERG.

#### 3.3.5. Analysis of the DEGs Involved in Signal Transduction Pathways

The responses to environmental signals are essential for the growth and development of microorganisms, and the signaling pathways currently well studied in fungi include protein kinase A/cyclic AMP (cAMP), protein kinase C (PKC)/mitogen-activated protein kinase (MAPK), lipid signaling cascades, and calcium-regulated neurophosphatase signaling pathways [41]. According to the KEGG pathways (Figure 5), DEGs related to the signal transduction were mainly enriched in the MAPK signaling pathway, followed by the phosphatidylinositol signaling system; however, no DEGs were related to the other signaling pathways under SMF compared with the CK.

The MAPK cascade is highly conserved as one key signal transduction pathway in fungi, plants and mammals [42], and can regulate a variety of cellular activities including cell proliferation, differentiation, survival, and death [43]. As shown in Supplementary Figure S2 and Table S7, compared with the CK, a total of 36 DEGs (28/8) and 18 DEGs (15/3) were found in the MAPK signaling pathway in the early (3 d) and late (7 d) growing periods, respectively. The results indicated that SMF could regulate the transcription levels of most genes in the pheromone response pathway, cell wall integrity pathway, high osmolarity pathway, and filamentous growth pathway relative to the MAPK signaling pathway in different culture periods. The sequential activation of the MAPK cascade may link the SMF stimuli with a wide range of cellular responses by activating downstream transcription factors [44].

Phosphoinositides (PIs), derived from phosphatidylinositol by phosphorylation, are key regulators of a large number of diverse cellular processes [45]. As shown in Supplementary Table S7, SMF ultimately affected the formation of the signaling lipids phosphatidylinositol-5-phosphate (PI5P) and phosphatidylinositol-4-phosphate (PI4P) by up-regulating the transcription levels of the *PIKFYVE* and *PI4KB* genes, as well as phosphate homeostasis by up-regulating the transcription level of *IPK1* [46,47].

#### 3.3.6. Effects of SMF on Transcriptional Factors

Transcription factors (TFs) are essential regulators of the gene expression in a cell [48] and play important roles in the signal transduction pathways, being the link between the signal flow and target genes [49]. The transcription levels of TFs under 30 mT SMF were analyzed, and the results are shown in Supplementary Table S8. We found that 20 (17/3) and 7 (3/4) TFs were influenced by SMF in the early (3 d) and late (7 d) growing periods. On the whole, SMF had a strong positive regulatory impact on TFs in the early growing period.

## 4. Discussion

Over the last few decades, numerous studies have revealed that MFs can virtually all affect living organisms, ranging from bacteria to human beings, [9,50,51,52,53]. However, the underlying mechanisms are still unclear [54,55], especially the impact of SMF on filamentous fungi. Therefore, we explored the effect of SMF on *M. ruber* M7 and its related molecular mechanisms. We found that SMF at 10 and 30 mT improved ex-CIT production (Figure 3). However, Wan et al. found that 1.6 mT LF-AMF reduced ex-CIT production [27]. Wang et al. reported that 10–35 mT of SMF exposure promoted the growth of *Chlorella vulgaris*, whereas 45 and 50 mT SMF had no effect on its growth [56]. So the influence of MFs on organisms may depend on different magnetic field types and intensities, action times, and the variability of cell structures [57,58]. This complexity may be one of the reasons for the consistent controversy of the effect of MF is positive or negative on various microorganisms [59].

This study found that the effects of SMF on MPs and CIT were mainly observed in the late (7 d) growing period, and, in particular, 30 mT SMF contributed to the accumulation of in-/ex-MPs and ex-CIT (Figure 3). Based on transcriptomic data, the levels of *MpigG* (serine hydrolase), *MpigM* (O-acetyltransferase), and *MpigA* (polyketide synthase) in the MPs gene cluster (Supplementary Table S6), as well as the genes (*MRL6*, *MRL5*, *MRL1*, etc.) in the CIT gene cluster (Supplementary Table S6), were significantly upregulated, which may contribute to the accumulation of MPs and CIT in the late growing period (7 d). In the early growing period (3 d), SMF significantly upregulated the transcript levels of most genes in the glycolysis/gluconeogenesis pathway (Supplementary Table S3), which can accelerate the conversion of starch and sucrose to pyruvate and thus contribute to the production of more biosynthetic precursors of MPs and CIT [60].

SMF also significantly upregulated most genes in the metabolic pathway of aromatic amino acids, such as phenylalanine and tyrosine (Supplementary Table S4), which might produce the biosynthetic precursors of MPs and CIT through a series of oxidation reactions [61,62]. Fatty acid degradation (Supplementary Table S5) was also enhanced, which contributed to the accumulation of acetyl-CoA, the biosynthetic precursors of MPs and CIT [63]. Overall, SMF may indirectly influence the accumulation of precursors for MPs and CIT synthesis mainly by positively regulating the transcript levels of genes related to the primary metabolic pathways in the early growing period (3d) and increase the yields of MPs and CIT by directly upregulating the transcript levels of genes in MPs and CIT biosynthetic gene clusters in the late growing period (7d).

**Figure 6:**
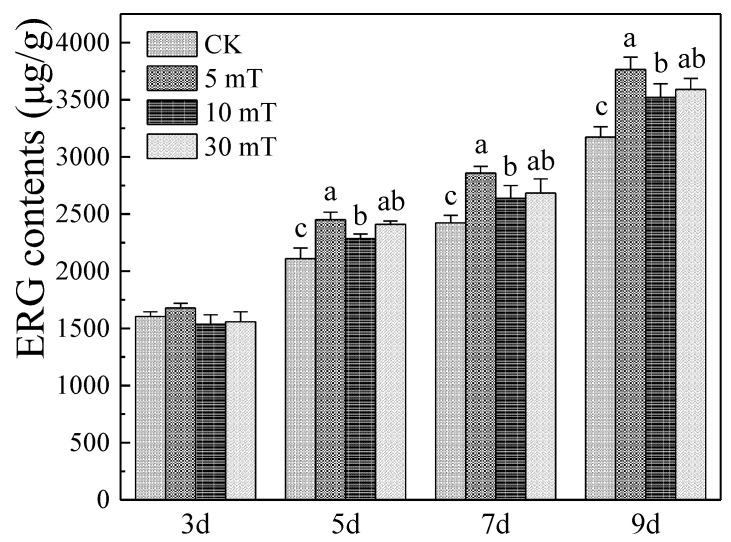
Ergosterol (ERG) produced by *M. ruber* M7 under SMF. The error bars indicate the standard deviations of three independent cultures. Lowercase letters signify a *p*-value < 0.05.

Furthermore, we found that SMF had a strong positive regulatory effect on ERG biosynthesis based on transcriptomic analysis (Supplementary Table S6). Therefore, we further explored the impact of SMF on the ERG content (Figure 6). The tested SMF contributed to the accumulation of ERG starting from day 5, and the ERG contents increased by 18.7%, 10.9%, and 13.2% at 5, 10, and 30 mT SMF at 9 d, respectively. Similarly, Romana et al. found that the weak LF-MFs contributed to the accumulation of membrane lipid ERG of *Pisolithus stinctorius* and speculated that the plasma membrane may be the receiver of the magnetic field signal [64]. The ERG-rich structures that exist on the fungal plasma membrane [65] contain proteins involved in cell signaling and stress response [66], and act as scaffolds to organize sensory components and, therefore, may be regulated by the MAPK signaling pathway [65,67]. Consequently, we speculated that the increase in ERG content may be influenced by the cell wall integrity pathway in MAPK cascades as a stress response to SMF stimulation.

In conclusion, the SMF (5, 10, and 30 mT) used in this study did not significantly affect the morphological characteristics of *M. ruber* M7. The 30 mT SMF contributed to the yields of in-/ex-MPs and ex-CIT, mainly in the late growing period (7 d). SMF had global impacts on M7 by activating MAPK cascades, especially on primary and secondary metabolism. Furthermore, SMF promoted an increase in ERG content, which may be a stress response to SMF stimulation. We expect that this work provides some clues to explain the mechanism of the SMF effect on *Monascus* spp. and other fungi.

## Figures and Tables

**Table 1:** Quality analyses of the transcriptomic data from *M. ruber* M7 treated under 30 mT static magnetic field (SMF).

Samples	Total Raw Reads (M)	Total Clean Reads (M)	Clean Reads Ratio (%)	Q20 (%)	Q30 (%)	GC (%)	Genome Mapping Ratio (%)	Gene Mapping Ratio (%)
CK-3d-1	47.33	44.25	93.50	97.20	89.22	52.50	95.96	78.94
CK-3d-2	45.57	42.58	93.43	97.20	89.22	52.10	95.94	78.49
30 mT-3d-1	45.57	42.26	92.72	96.36	87.67	53.20	94.83	78.13
30 mT-3d-2	45.57	42.34	92.91	96.30	87.49	53.70	95.10	78.77
CK-7d-1	45.57	42.92	94.17	97.08	88.95	53.30	95.91	76.99
CK-7d-2	47.33	44.10	93.19	97.19	89.20	53.00	95.54	76.88
30 mT-7d-1	47.33	44.21	93.40	97.05	88.74	53.20	95.56	77.18
30 mT-7d-2	47.33	44.47	93.97	97.29	89.55	52.70	96.09	78.01

## Data Availability

The raw data supporting the conclusions of this manuscript will be made available by the authors, without undue reservation, to any qualified researcher.

## Supplementary Materials

The following are available online at https://www.mdpi.com/article/10.3390/jof7040256/s1, Figure S1: Morphological characters of *M. ruber* M7 under SMF, Figure S2: DEGs in the mitogen-activated protein kinase (MAPK) cascade of *M. ruber* M7 under 30 mT SMF, Table S1: Primer information of genes in qRT-PCR verification, Table S2: KEGG pathway enrichment DEGs of CK-7d vs 30 mT-7d, Table S3: Differentially expressed genes (DEGs) related to carbohydrate metabolism, Table S4: DEGs related to amino acid metabolism, Table S5: DEGs related to lipid metabolism, Table S6: DEGs related to secondary metabolism, Table S7: DEGs related to Signal transduction pathways, Table S8: DEGs of transcription factors.
