# Supplementary material for: Effect of Static Magnetic Field on Monascus ruber M7 Based on Transcriptome Analysis
Source: J Fungi (Basel). 2021 Mar 30;7(4):256. doi: 10.3390/jof7040256 (PMC8066190; doi:10.3390/jof7040256)
Supplement: Supplementary file 1 [file jof-07-00256-s001.zip › jof-1163320-supplementary/Supplementary materials/Figure S1. Morphological characters of M. ruber M7 under SMF.docx]

**
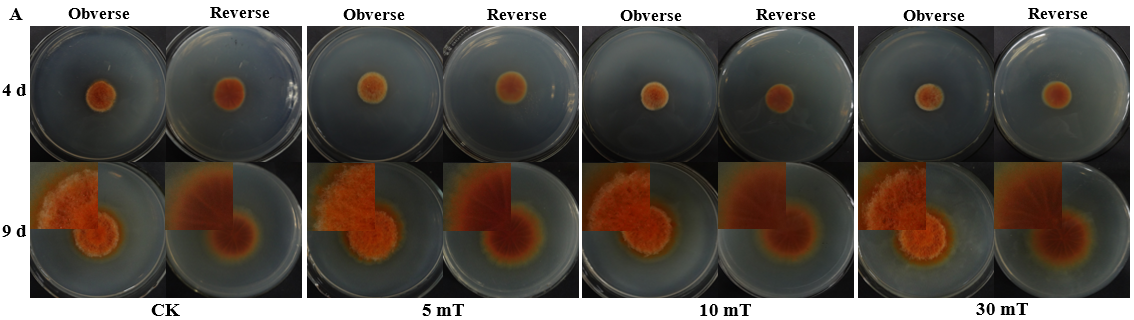
**


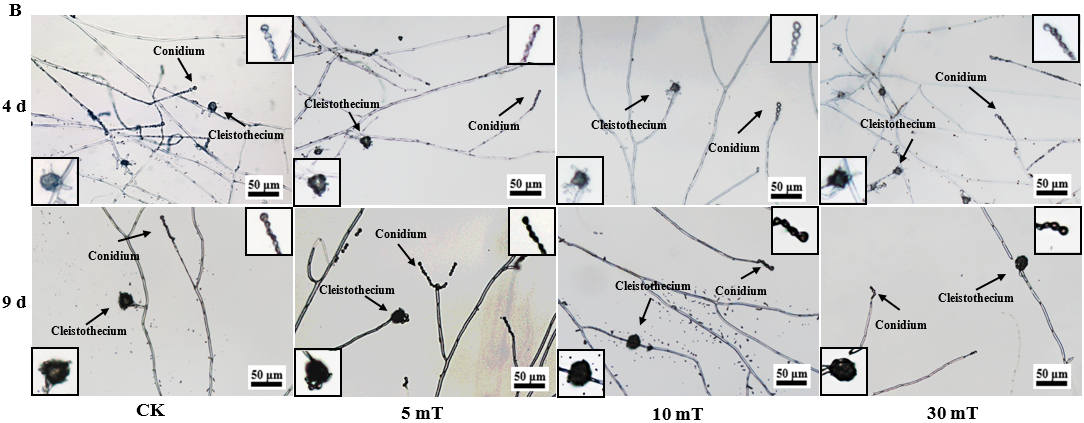


**Figure S1. Morphological characters of *M. ruber* M7 under SMF**

A. Colonic morphologies of M7; B. Microscopic structures of M7.

The original images are as follows:


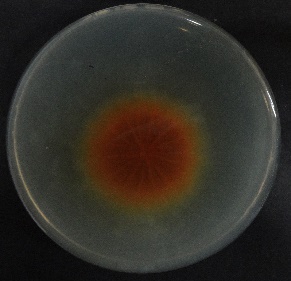

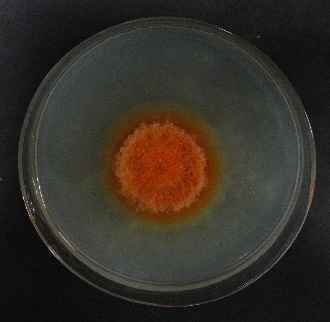

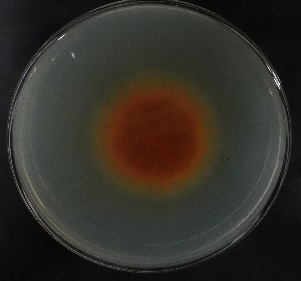

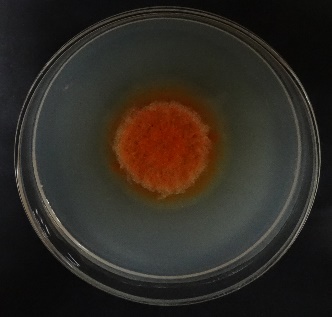

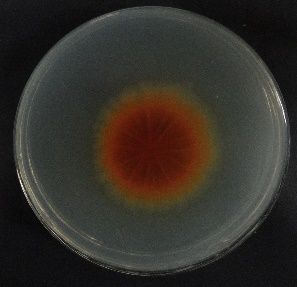

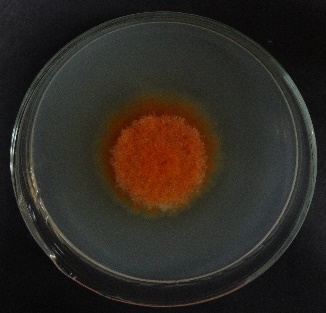

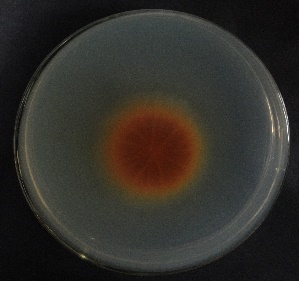

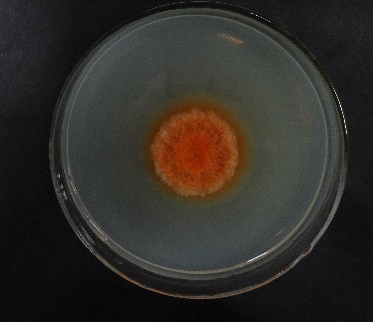


**Obverse**

**CK**

**5 mT**

**10 mT**

**30 mT**


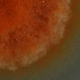

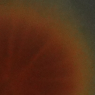

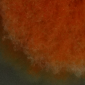

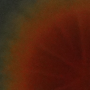

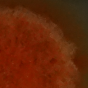

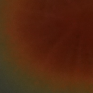

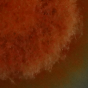

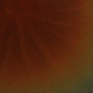


**Reverse**

**Obverse**

**Obverse**

**Obverse**

**Reverse**

**Reverse**

**Reverse**

**4 d**

**9 d**

**A**


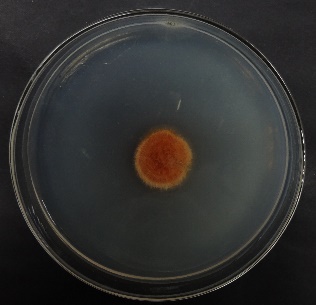

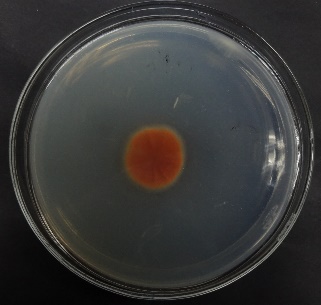

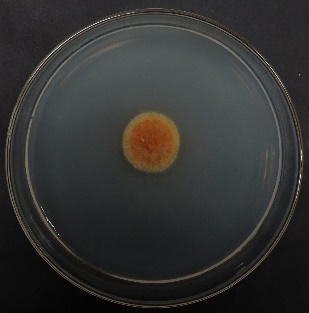

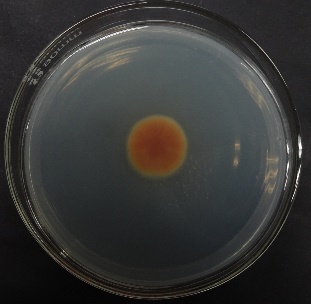

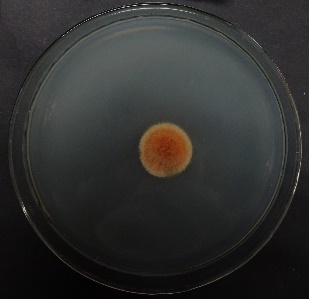

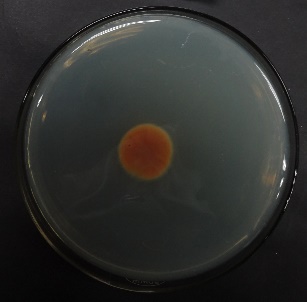

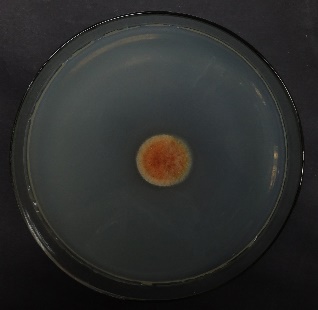

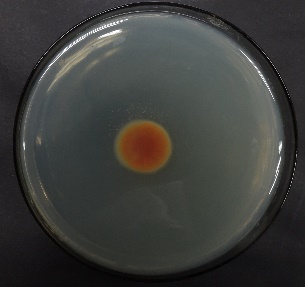

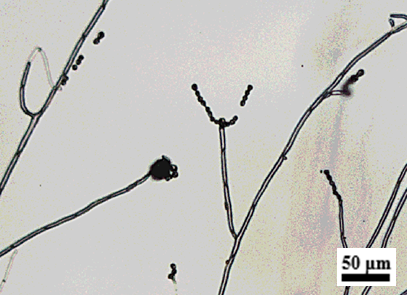

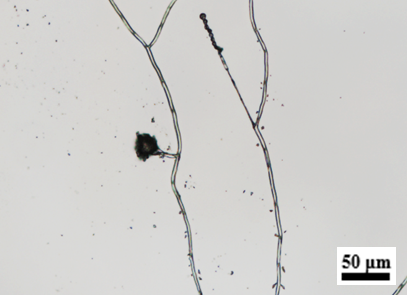

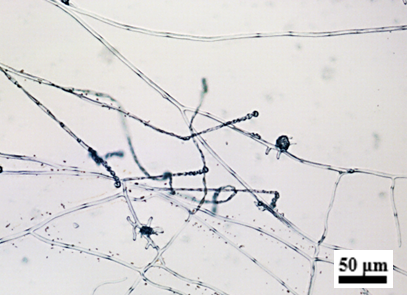


**Cleistothecium**

**Conidium**

**4 d**

**Conidium**

**Cleistothecium**

**CK**

**9 d**


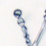

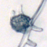

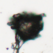

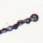

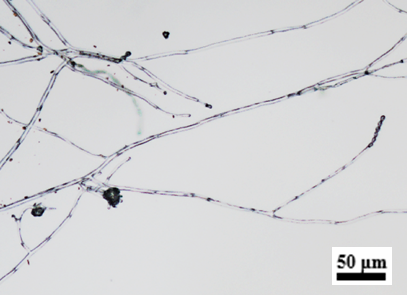


**Conidium**

**Cleistothecium**

**5 mT**

**Conidium**

**Cleistothecium**


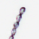

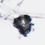

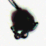

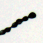

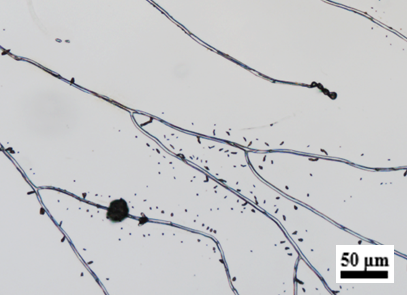

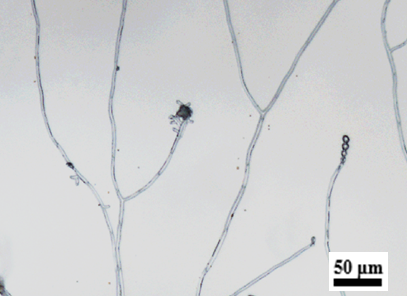


**Conidium**

**Cleistothecium**

**10 mT**

**Conidium**

**Cleistothecium**


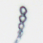

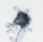

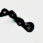

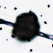

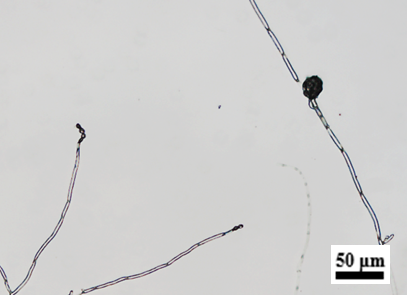

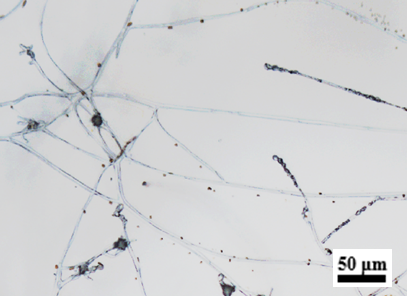


**Cleistothecium**

**Conidium**

**30 mT**

**Conidium**

**Cleistothecium**


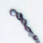

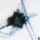

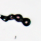

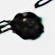


**B**
