# Supplementary figures and images for: Effect of Static Magnetic Field on Monascus ruber M7 Based on Transcriptome Analysis
Source: J Fungi (Basel). 2021 Mar 30;7(4):256. doi: 10.3390/jof7040256 (PMC8066190; doi:10.3390/jof7040256)

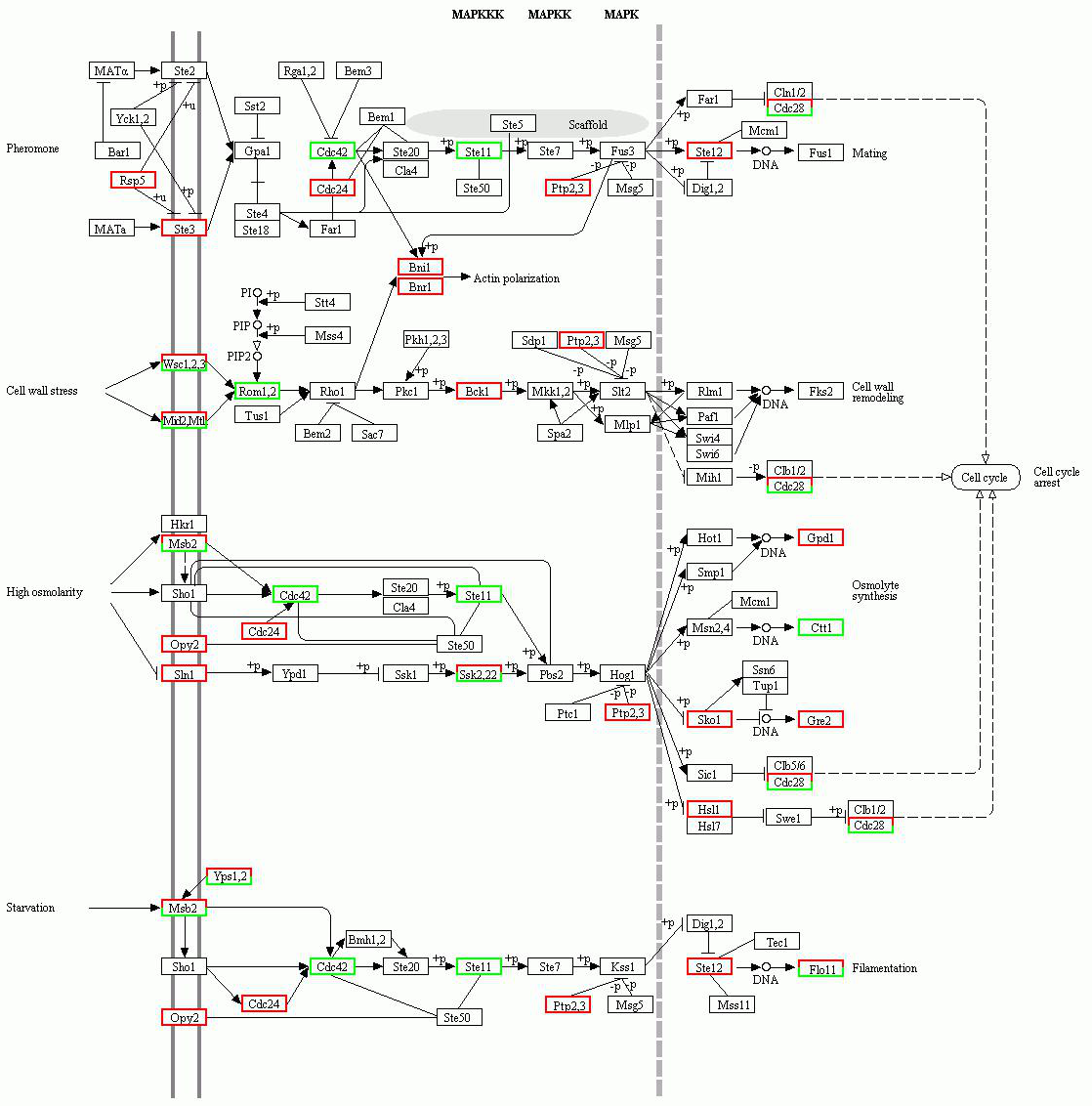

Supplement: Supplementary file 1 [file jof-07-00256-s001.zip › jof-1163320-supplementary/Supplementary materials/Figure S2. DEGs in the mitogen-activated protein kinase (MAPK) cascade of M. ruber M7 under 30mT SMF A.CK-3d vs SMF-3d Red upregulated; green downregulated..png]

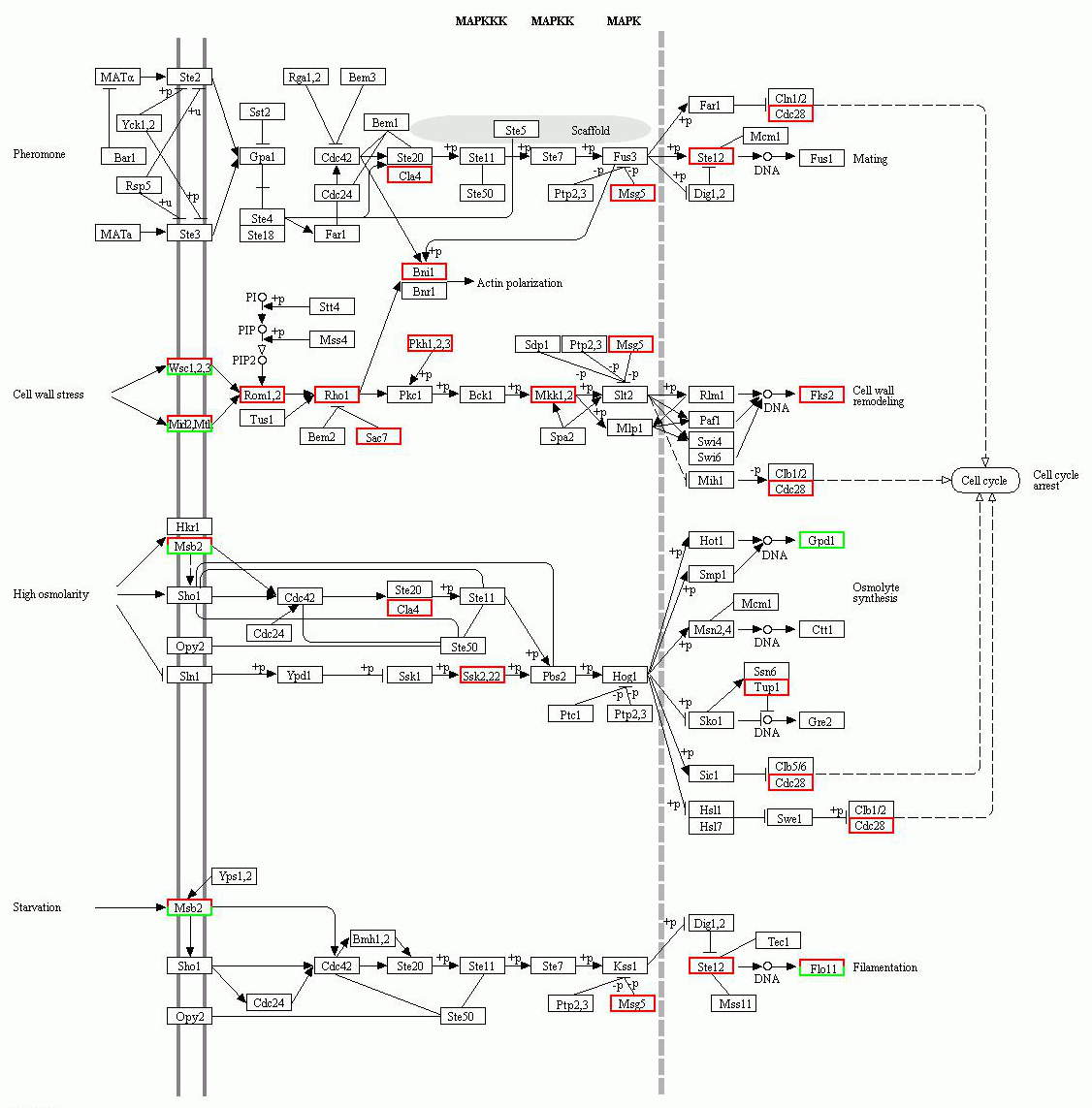

Supplement: Supplementary file 1 [file jof-07-00256-s001.zip › jof-1163320-supplementary/Supplementary materials/Figure S2. DEGs in the mitogen-activated protein kinase (MAPK) cascade of M. ruber M7 under 30mT SMF B. CK-7d vs SMF-7d.png]

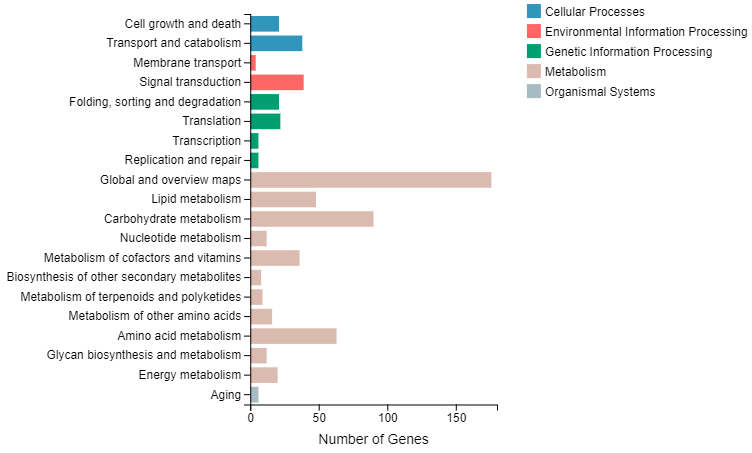

Supplement: Supplementary file 1 [file jof-07-00256-s001.zip › jof-1163320-supplementary/Supplementary materials/The original images of Figure 5/Figure 5 (A).jpg]

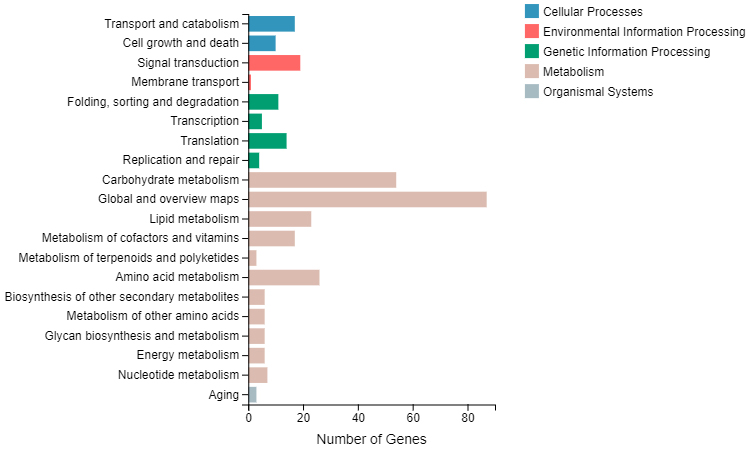

Supplement: Supplementary file 1 [file jof-07-00256-s001.zip › jof-1163320-supplementary/Supplementary materials/The original images of Figure 5/Figure 5 (B).jpg]

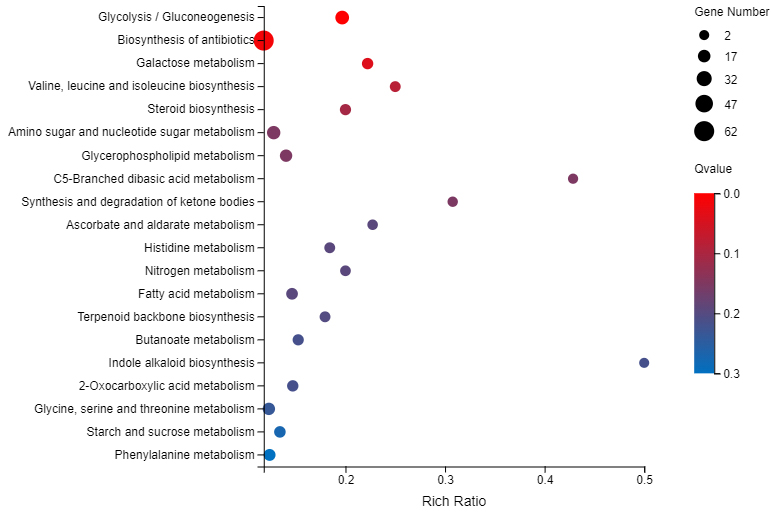

Supplement: Supplementary file 1 [file jof-07-00256-s001.zip › jof-1163320-supplementary/Supplementary materials/The original images of Figure 5/Figure 5 (C).jpg]
